# Paediatric oncology in the Eastern Mediterranean region (EMR): the current state and challenges

**DOI:** 10.3332/ecancer.2024.1677

**Published:** 2024-02-28

**Authors:** Arsalan Kabir Siddiqui, Asim Fakhruddin Belgaumi

**Affiliations:** Department of Oncology, Aga Khan University, Karachi 74800, Pakistan; † All authors have contributed to this manuscript and take responsibility for its contents.; a https://orcid.org/0000-0002-0135-7509

**Keywords:** childhood cancer, Eastern Mediterranean region, Middle East, paediatric, oncology, children

## Abstract

The WHO Eastern Mediterranean region (EMR) is characterised by highly economically diverse countries, with healthcare systems in various phases of development. Childhood cancer care provision also ranges from that provided in centres able to deliver sophisticated therapy resulting in outcomes comparable to those seen in highly developed nations, to countries with no provision for care of children with cancer. At 10·1 per 100,000 children at risk, the age standardised incidence-rate for cancer in children below 14 years of age is relatively low but may be consequent to poor registration. Shortages in trained care providers were identified in many regional countries, particularly in low and lower-middle income countries, however, implementation of training programs are beginning to counter this deficit. Significant diversity in patient care capacity exists in the region, leading to inequitable access to quality paediatric oncology care. There is strong potential for regional collaboration towards infrastructure and capacity improvement, with facilities available within the EMR for twinning and educational support to those centres and countries that need them. While cancer care coverage is available to citizens of high-income countries, in the lower-income countries out-of-pocket health expenditure can reach 75%. Some relief is achieved through the contribution of multiple charitable foundations working to support childhood cancer care in the region, as well as the provision of care in, often overburdened, public sector hospitals. War and other geo-political turmoil, as well as natural disasters, have negatively impacted healthcare capacity, including childhood cancer care, in several regional countries. Despite all this, the trajectory for change is upward and initiatives such as the WHO Global Initiative for Childhood Cancer are igniting positive change.

## Introduction

**Figure 1.**
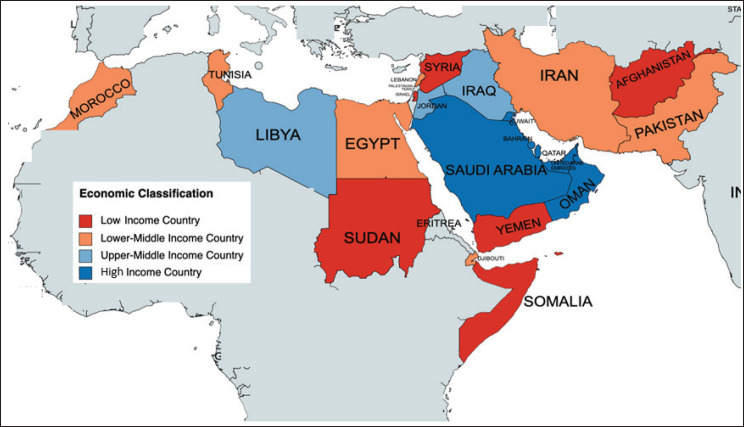
Map showing the countries that constitute the EMR. The countries are colour coded according to their World Bank economic status.

Comprising close to 745 million people, the WHO-delineated Eastern Mediterranean Region (EMR) is one of incredible heterogeneity. It consists of 21 member states, 14 in Asia and 7 in Africa, as well as the Occupied Palestine Territory (OPT) [1] (See Figure 1). A variety of local languages are used in the region, most commonly Arabic, Urdu, English and Farsi [2]. This diverse region includes high-income countries (HIC) like Saudi Arabia, Qatar and United Arab Emirates (UAE) along with low-income countries (LIC) like Somalia, Sudan and Yemen [3]. The contrasting nature of the region is further reflected in the gross domestic product (GDP) per capita, which ranges from $368 to $66,838 per capita [4]. As of the most recently available data, more than 25% of the population is living below the international poverty line in six EMR countries [5]. Similarly, other indices such as infant mortality rate and percentage of the population with access to electricity range from 5 to 73 per 1,000 live births and 47.3%–100%, respectively [6, 7] This variability in socio-economic conditions has also impacted levels of investment in healthcare. While there has been a progress in childhood cancer care in most of the regional countries, the trajectory has varied, with centres of excellence in some countries, while others continued to have meagre care capacity [8].

Another defining feature is the number of expatriates in many EMR countries, both due to economic motivations and, in many cases, necessity given socio-political conflicts. For example, expatriates make up 88.4% of UAE’s and 32.3% of Saudi Arabia’s population and refugees make up nearly 20% of Lebanon’s population [9, 10]. For many EMR countries, the rapid influx of non-citizens has strained health systems and has impacted healthcare delivery.

As global efforts have improved healthcare access and reduced the burden of communicable diseases, childhood cancer is becoming a leading cause of death in the paediatric population. An estimated 400,000 children are diagnosed with cancer annually, with 90% of these cases occurring in low and middle income countries (L/MIC) [11]. Alarmingly, only 20% of children diagnosed with cancer in these countries will survive their disease [12]. Additionally, as children make up a larger proportion of the population in L/MICs, this potentially results in a higher load of childhood malignancies for healthcare systems to handle [13].

Countries in the EMR must contend with many challenges that impact the treatment of children with cancer. Eight EMR countries are currently facing conflict and humanitarian emergencies, and over 127.3 million people require humanitarian assistance in the region, as of 2022 [14]. Given the rising significance of childhood cancer, it is imperative to delineate the current state, the challenges faced, and the avenues of improvement for childhood cancer treatment in the EMR. The objective of this review article is to provide a comprehensive overview of the status of childhood cancer care in the region, particularly emphasizing the variability in quality and scope of services, and the socio-economic pressures affecting the provision of care. The article also discusses the opportunities available in the region for improvement, particularly through collaboration.

## Methods

A literature search was conducted through PubMed^R^ (pubmed.ncbi.nlm.nih.gov) for published articles referencing any of the 22 member countries and territories. Additional terms, such as ‘Middle East’, ‘Arab’, ‘Arabian’, ‘Northern Africa’, were also utilised. These were further filtered using search terminology for cancer (e.g., cancer; malignancy; malignancies; tumor; tumour; etc.) and the paediatric age group (e.g., child; children; young; pediatrics; paediatrics; etc.).

These publications were read by both authors and categorised according to the areas of focus. Where similar information was available in more than one publication that from the most recent publication was used. Additional sources of information relating to national socio-economic parameters, healthcare indicators, and cancer care regionally or globally were also utilised, and included published articles, websites and publicly accessible databases. As this was not a meta-analysis or systematic review, the inclusion of information and sources was not formally structured.

### Cancer incidence and burden

In 2022, the first comprehensive summary of the documented childhood cancer burden in the EMR was published [15]. Although Fadhil *et al* [15] acknowledged that they may have underestimated the true burden, their findings remain insightful. The age standardised incidence-rate in the children below 14 years of age was 10·1 per 100,000 children at risk. This incidence was lower than those reported from the European, Americas and Western Pacific regions where cancer registration is well-established. Similarly, EMR countries with national level cancer registries (e.g., Saudi Arabia, Lebanon, and Iran) and those with strong referral systems (e.g., Morocco, and Egypt) report higher childhood cancer incidence. This may indicate a suboptimal incidence estimate, particularly in largely populated countries like Pakistan. While the age-standardised incidence rates are similar or lower than that seen in other WHO regions, there is a higher number of estimated incidents (23,847 in 2020) due to the relatively greater paediatric population in the EMR [15]. More significantly, at 4·4 per 100,000 children at risk, the age-standardised mortality rate for childhood cancers in the region was the second highest among all WHO regions [15]. This can be an indicator of the limited capacity and resources to effectively treat children with cancer in some countries, as will be discussed below. Furthermore, a negative correlation was found between the country’s GDP per capita and the age-standardised mortality rates, with lower mortality rates in the HIC [15]. Pakistan and Egypt, the two countries with the highest populations in the EMR, accounted for more than 40% of all incident childhood cancer cases and the highest burden of childhood cancer mortality, with 41% of all deaths occurring in these two countries.

Population-based cancer registries (PBCRs) are the gold standard in cancer surveillance systems [16]. It allows one to quantify the burden, effectiveness of treatment and outcomes of cancer patients in one’s setting [16]. According to the International Agency for Research on Cancer, PBCRs are developing slower in weaker-economy countries compared to stronger economy countries due to underinvestment despite the awareness of its importance [17]. Of the 22 EMR members, 6 of 9 HIC/upper-middle income countries (UMIC) and 3 of 13 lower-middle income countries (LMIC)/LICs have national level PBCRs [18]. However, the latest published data from these countries was in 2012, and a more updated audit is needed [18]. Eight countries have sub-national PBCRs with variable functionality, while five countries have no registries (Table 1). Along with financial limitations, the limited capacity of PBCRs in the EMR is also contributed to by political and social challenges. Armed conflicts have halted cancer registration in Syria, the only LIC with a national-level registry, while the sub-national registries in Yemen and Libya are currently discontinued [19]. In others, population shifts due to conflict and disasters have complicated the production of quality data for cancer care [19].

The quality of registration and inclusion of the paediatric population varies from country to country [15]. Although Lebanon has had a national cancer registry since 2005, its quality has been subpar and patient registration has been inconsistent. In addition, the registry inflates the number of registered cancer cases relative to Lebanon’s population as it includes those seeking consultation from neighbouring Syria and Iraq [20]. In contrast, the Gulf Center for Cancer Registration, which publishes all cancer registrations of Oman, UAE, Saudi Arabia, Kuwait and Qatar, is considered accurate, but also only provided a snapshot from 1998 to 2007 [21]. In Jordan, the national PBCR lacks data on patient outcomes, and data of paediatric patients up to only 15 years of age [20]. Similarly, only about 5,000 new patients can be accounted for at the various subnational PBCRs in Pakistan, suggesting that a large proportion remain undiagnosed or not accounted for (GLOBOCAN 2020 estimates the incidence cases of 5574) [20].

In 2020, the WHO released Cancer Country Profiles which aimed to synthesize the status of cancer control for each WHO Member State. Presenting data from multiple sources, it includes information like cancer burden, trends and health system capacity in each member state. Table 1 below highlights some key findings from each country.

### Healthcare manpower

The WHO recommends a ratio of 10 physicians per 10,000 individuals, which, on average, the EMR meets with 12 physicians per 10,000 patients [22]. However, this ratio varies between countries. Countries with higher resources like Jordan (26.6), Qatar (24.9) and Saudi Arabia (27.4) have achieved this ratio, whereas it is subpar in countries like Egypt (2), Morocco (7.3), Djibouti (2.2), Afghanistan (2.5) and Yemen (5.3) [22]. In Pakistan, one paediatric oncologist may see over 100 new patients per year which is twice the *Lancet Oncology* Commission recommendation of one paediatric oncologist for every 50 newly diagnosed patients in low and lower-middle income countries (L/LMICs) [20, 23].

Unfortunately, data in the EMR on the healthcare workforce involved in paediatric oncology is limited. The Pediatric Oncology East and Mediterranean (POEM) Group, which includes representatives from a majority of EMR countries, recently published a report from its member centres on the state of the workforce and training needs of the group [24]. At the 50 (of 82) member centres that responded to the study, there were 299 trained paediatric oncologists, with median of 4 at each centre. The proportion of new cancer patients to paediatric oncologists was found to negatively correlate with GDP per capita; ratios in HIC were like those found in USA or Europe. Additionally, 255 physicians without formal PO training participated in cancer care in these centres. Similarly, nurses with general paediatric training constituted the largest number of providers for PO patients; this was unrelated to country income level. A smaller number of general nurses also supported childhood cancer.

Many paediatric oncologists (40.8%) received training abroad, in large part due to the limited local postgraduate opportunities [24]. In Jordan, a UMIC, a majority of their 20 paediatric oncologists in 2012 received post graduate certification in the US, UK and Europe [25]. Similarly in Morocco, paediatric oncologists receive postgraduate training in French-speaking countries [26]. Although it is still insufficient to meet the demands of the region, many countries in the EMR provide PO fellowship training for physicians. Some countries like Saudi Arabia and Pakistan have established mandatory national board examinations for paediatric oncology to be categorised as a subspecialist [8]. This has resulted in a considerable increase in the number of trained paediatric oncologists in these countries. Initiatives by the POEM group and the African School of Paediatric Oncology (Ecole Africaine d’Oncologie Pediatrique, EAOP) have opened opportunities for short-term training at major cancer centres in their respective regions for both physicians and nurses [27].

In their report, the POEM group further found that 9 centres offered PO training for both physicians and nurses, 16 only for physicians and 4 for nurses only, and 21 centres lacked any PO training programs. The availability of PO physician training opportunities was found unrelated to country income. Formal PO nurse training centres in nine countries had a total capacity to train 272 nurses each year. None were in LICs where the need is probably greatest. The proportion of surveyed centres with PO nurse training programs was highest in HICs (44.4%), and these countries also had the highest number of foreign-trained PO nurses [24].

### Childhood cancer care facilities

Facilities for childhood cancer care vary considerably in the region. These range from advanced care facilities in dedicated children’s cancer centres and multi-specialty tertiary care hospitals, to countries with no resources to treat children with cancer. The comprehensiveness of effective paediatric cancer treatment requires a multidisciplinary approach, which is considered a chief driver of improving childhood cancer morbidity and mortality over the past 30 years [28]. These modalities include the capacity for providing surgery, chemotherapy, radiotherapy and palliative care for PO patients. Unfortunately, this multidisciplinary approach is most often only found in advanced healthcare system within the countries.

In the US, there are 50 NCI-designated paediatric centres which can provide cutting edge, specialised care in paediatric oncology [29]. In contrast, there are only four dedicated paediatric cancer centres in the EMR: the Children’s Cancer Hospital of Egypt (CCHE), the King Fahad National Center for Children’s Cancer and Research (KFNCCC), Lebanon’s Children’s Cancer Institute (CCI), and Iran’s Mahak Hospital and Rehabilitation Complex. KFNCCC cares for a third of all paediatric cancers in Saudi Arabia, CCHE cares for half of its country’s cases, and the

CCI sees approximately 40% of cases in Lebanon [20, 30]. Most countries have cancer centres catering to care for all ages, like the King Hussein Cancer Center, which treats approximately 450 PO patients (approximately 70% of total cases) yearly, and Shaukat Khanum Memorial Cancer Hospital and Research Centre in Pakistan, which treats 550–650 paediatric patients annually [20, 31]. Within these well-established cancer care centres, the high-quality care patients receive resembles other advance healthcare systems. In other instances, childhood cancer is treated at advanced tertiary care centres which provide the multidisciplinary approach required in the care of PO patients. However, these centres are often able to support a small proportion of children with cancer in their country. The Aga Khan University Hospital in Pakistan, for example, annually treats about 200 new cases of cancer in patients younger than 18 years of age, constituting only 4% of diagnosed childhood cancer cases across Pakistan [20].

In many EMR countries, most PO patients are seen at general paediatric centres. In Baghdad, Iraq, the Children’s Welfare Teaching Hospital accepts an average of 300 PO cases annually that are admitted to a paediatric oncology ward within the hospital [20]. Similarly, in Pakistan several public children’s hospitals have PO units that provide care to over 2,000 children with cancer (AB Personal information). Although an estimate of the total burden of paediatric cancer in these countries is unclear, it is fair to assume that a significant proportion are either referred or treated at these hospitals. In general, these hospitals are understaffed and over-burdened, and are slow in adopting new technology and medications due to resource limitations.

The capacity to deliver care in government-run general hospitals varies considerably. In HIC, centres such as the King Fahd Medical City are well-funded and resourced and can provide high-quality paediatric oncology care. The range of quality-of-care provision in public hospitals in LIC/LMIC is large, with reasonably resourced centres such as the Children’s Hospital, Lahore, able to diagnose and treat large numbers of patients utilising a robust, multidisciplinary approach at one end and hospitals with poor provision of resources and technology, such as the National Oncology Center in Sana’a, Yemen, on the other end [32]. In EMR countries like Somalia and Djibouti, paediatric cancer care is almost non-existent [32].

As is evident, significant diversity in patient care capacity exists in the region, leading to inequitable access to quality paediatric oncology care. There is strong potential for regional collaboration towards infrastructure and capacity improvement, with facilities available within the EMR for twinning and educational support to those centres and countries that need them.

### Diagnosis and treatment

While the referral and care provision for children with cancer within this region has improved significantly, there remain multiple areas for improvement. Delays in referral and late presentation remain an issue across most countries in the region [20]. There has been some increase in awareness both amongst the public and the medical community, which has resulted in some anecdotal improvements in presentation delays; this however needs to be confirmed (AB personal information). Additionally, clear referral pathways need to be established in many countries in the region. Saudi Arabia has had centralised cancer care delivery at dedicated tertiary care centres for decades. While systems to refer patients to these centres had been paper-based and patient-dependent, over the past several years much investment in this has been made and efficient electronic systems are now available at the Ministry of Health (MoH) level and for some individual hospitals [33]. The ‘Ehalati’ referral system introduced by the Saudi MoH is a comprehensive referral system for all primary to secondary to tertiary referrals across the Kingdom, and although shortcomings were reported in earlier evaluations, improvements seem to be continuing [34]. Directed cancer referral systems to comprehensive cancer care centres have also been implemented; one such for the Princess Norah Cancer Center in Jeddah has demonstrated efficacy [35]. Similar systems exist in Qatar, with referrals from their Primary Health Care Centers to the referral services. A recent publication has documented the pre-diagnosis symptom interval for children with brain tumours to be acceptable, but with opportunities for further improvement identified [36]. A similar study from Morocco noted a significant delay in diagnosis for paediatric brain tumours, with a median time to diagnosis of 2 months [37]. With almost half of all childhood cancer in Egypt being cared for at the CCHE the demand for referral and admission often results in delay, with potential for advanced stage and poor outcomes. Even though treatment centres are present, formal referral systems are not available in many regional countries, such as Pakistan and Lebanon [20].

Over the past decade, significant advancement has occurred in diagnostic capability. All the major centres can perform most of the required basic diagnostic and staging studies, such as flow-cytometry, immune-histochemistry, computerised tomography or magnetic resonance imaging [8]. In most countries and cities, there are resources available for referral of patients or samples for some of the more specialised tests, such as cytogenetics or molecular genetics, and positron emission tomography scan(s). While the required diagnostic tools (e.g., imaging modalities, immunohistochemistry, flow-cytometry and cytogenetics) are largely available in major cancer centres, the educational and experiential capacity for conducting comprehensive diagnoses may vary and/or be lacking in different centres even within the same country [8]. This has resulted in the development of regional twinning programs to help build local capacity. One such program implemented by the POEM group was consequent to decline in pathology expertise in Iraq and Syria following the years of conflict (AB Personal information). This involved secondary review of paediatric oncology pathology specimens by pathologists at the American University of Beirut, with collaborative reporting of the findings. This allowed for improved diagnostic quality as well as engendered learning and expertise development among pathologists in the recipient countries (AB Personal information).

The general availability of chemotherapy in the public sector is a WHO country level indicator that documents whether the country has reported that chemotherapy is generally available in the public sector [38]. ‘Generally available’ is defined as reaching 50% or more patients in need. Of the EMR countries three (Djibouti, Somalia and Syria) reported not achieving this indicator for 2021, while responses from Egypt (likely achieved) and Yemen (likely not achieved) were not known [38]. Palestine was not included in the list. The WHO lists 42 medicines used in oncology treatment as essential for children in 2021[39]. In a survey on the availability of anti-neoplastic agents included in the 2015 WHO’s List of Essential Medicines for Children in non-European countries, it was found that while most medications are available to patients at either no cost or subsidised in participating HIC and UMIC EMR countries like Saudi Arabia, Iran, Qatar, Oman, UAE and Iraq, other EMR countries that are LMIC, like Pakistan and Afghanistan, only have them available at full cost [40]. Moreover, the essential anti-neoplastic agents were not always available in Lebanon, Morocco, Iraq, OPT and Afghanistan [40]. Some nongovernmental organisations in the region have taken the responsibility for providing anti-cancer medications in their respective country, such as in Morocco where the Lalla Salma Association Against Cancer provides medication to all government run-oncology units in the country [26]. Institutions such as the King Hussain Cancer Center have opted to import their required medications directly from international manufacturers to avoid loco-regional shortages (AB Personal information).

Regarding therapeutic modalities in the EMR, the scarcity of radiotherapy machines is another resource limitation. Based on the Group II WHO recommendations of one megavoltage machine per one million population, the EMR would need 745 mega voltage machines (MVM) at a minimum for cancer treatment in their region [41]. According to the Directory of Radiotherapy, EMR countries currently possess 512 MVMs, approximately 68% of the region’s needs [42]. Although many of the EMR countries meet the WHO recommendations, facilities are not equally distributed. Adequate MVM numbers are seen in countries with better medical facilities, such as Jordan, UAE, and Lebanon while severe shortages exist in Syria, Iraq and Yemen. In Iraq, for example, waiting lists for paediatric patients that require radiotherapy can be up to six months [43]. In Pakistan, additional to the radiation oncology facilities available in some private and public hospitals, the Pakistan Atomic Energy Commission, a federally funded independent government body, has established 19 cancer treatment centres spread over the country, that are focused on the provision of radiation treatment either free of charge or at subsidised rates [44]. Although the focus and expertise in these centres is predominantly adult-centric, and the capacity and quality of treatment they provide is highly variable, these remain a potential resource for paediatric radiation oncology referral.

### Cost of treatment – the patient’s burden

In many HICs of the EMR like Saudi Arabia, UAE and Kuwait, all citizens have universal healthcare coverage, including cancer care. Similarly, in Jordan and Bahrain, cancer care is free or subsidised [45]. This assures that the financial limitations do not play a role in the challenges of PO treatment for citizens in these countries. For non-citizens in these countries however, healthcare is dependent on private insurance plans [8]. In the lower-income countries of the EMR, the WHO reports that out-of-pocket health expenditure can reach 75% [46]. In Egypt, Pakistan, and Afghanistan, the cost of PO treatment is documented as difficult for many families to afford, and it can lead to job abandonment and neglect of other children, especially as the average family size in the EMR countries is around five [32]. Similarly, transportation and accommodations can be a major challenge to the continuity of care [32]. These difficulties lead to the relatively higher rates of treatment abandonment in the region. Studies from Morocco have shown that one-third children abandon rhabdomyosarcoma treatment, 12.5% abandon Hodgkin Lymphoma treatment, and one in five patients abandon Wilms tumour treatment, which impacts the overall event-free survival of these malignancies in the country [47–49]. Similarly in Iraq, 11% of high-grade non-Hodgkin lymphoma paediatric patients abandon therapy, causing lower outcome amongst these patients [50]. In Pakistan, abandonment rates are highest in patients with brain and solid tumours [20]. Abandonment was associated with socio-economic status and distance from treating centre in a study of ALL patients in Karachi, Pakistan, even when the direct cost of treatment was covered, underscoring the impact of indirect costs, such as days away from work and travel expenses [51]. In some EMR countries, charitable hospitals like the CCHE and Mahak Hospital and Rehabilitation Complex have financially supported a significant number of patients, enabling access to care at advanced healthcare settings. Even so, these charities are still not sufficient for a vast majority of PO cases in their respective countries [8].

### Conflict and natural disasters

Ever since the Arab Spring, a movement of pro-democratic revolts, started in 2010–2011, the ensuing political instability and regional conflicts has fundamentally altered the state of healthcare in many EMR countries. In 2022, 245 attacks on healthcare facilities were recorded in seven EMR member states [14]. Only 50% of the pre-war hospitals and health facilities were functional in 2020 because of the 2014 Yemen civil war [52]. Many of the prominent countries in the Arab Spring, like Egypt, Syria and Libya, were showing significant improvements in its health indicators until the 2010s. Between 1990 and 2013, for example, there was a 62.5% reduction in maternal mortality rates in Egypt [53]. Prior to its 2011 civil war, Libya was the highest ranked African country in the Human Development Index and nationals received universal health coverage [54]. Similarly, in the Syria Arab Republic, infant mortality rates reduced from 132 to 17.9 per 1,000 between 1970 and 2009, and life expectancy had shown an average of 0.25-year increase per year between 1990 and 2010 [55]. However, by 2013 as a direct result of the conflicts in the region, the life expectancy had dropped 0.25 years in Egypt, Tunisia and Yemen since 2010 [56].

War-associated injuries impose a heavy burden on a country’s healthcare system. Local hospitals become overloaded and life-threatening injuries are prioritised over ‘regular’ patients. In Syria, this has led to a neglect of a large proportion of patients with chronic conditions [55]. Moreover, clinical workers suffer heavy emotional stress due to increased working hours and lack of safety while working [57]. Within this context, it is almost inconceivable to receive childhood cancer care in one’s country. Many in these crisis-filled countries have sought asylum in neighbouring EMR countries like Lebanon, Jordan, Pakistan, Iran and Egypt. This has stressed their respective healthcare systems, as most refugees do not have healthcare coverage. Iran hosts over 8 million refugees from Afghanistan stretching the capabilities of Iran’s otherwise well-developed health systems and costing the Iranian government $32 million per year [14]. Similarly, of the 6.7 million Syrian refugees, 1.5 million are in Lebanon, more than 665,000 are in Jordan, and close to a quarter of a million are in Iraq [58]. The impact of this refugee crisis on cancer care has also been well-documented. In Egypt, for example, patients from Lebanon, Yemen and Syria are increasingly presenting for diagnosis and treatment, with little data on their numbers, treatment outcomes, and financial costs [20]. Mansour *et al* [59] reported the cost of providing 900 Syrian refugees (both adult and paediatric) oncological treatment in Jordan was approximately 22 million USD. Similarly, Saab *et al* [60] reported that 575 Syrian refugee children were treated at the CCI in Lebanon over 2001–2017. Due to the continued lack of facilities in their own country, patients from Afghanistan continue to come to Pakistan for treatment, extending the pressure on an already stretched system [8, 61]. Other than the resource-impact refugees may have on host countries, they may also have worsened outcomes relative to locals. A Turkish study found that, although cancer care is financially covered for them, Syrian refugees had significantly lower survival rates independent of cancer type or stage [62]. The authors believe this may be due to measures needed to establish initial care, leading to delayed presentation in this population [62].

The EMR has recently been devastated by the Turkey-Syria earthquakes in February 2023. It has been one of the largest recorded natural disasters in the region, impacting more than 4.5 million Syrians citizens [63]. It has damaged more than 55 health facilities, exacerbating the already weakened health system that was unable to meet the needs of the region [63, 64]. This, however, has not been the only recent natural disaster in the EMR. Between June and October 2022, massive rains and melting glaciers has led to 6.4 million people in Pakistan requiring humanitarian health assistance in the end of 2022 [65]. In Somalia and Djibouti, a persistent drought has left close to 1.1 million Somalians and 400,000 Djiboutians requiring humanitarian assistance respectively [14]. These types of natural disasters have devastating effects on the health infrastructure throughout the region. Hospitals can become quickly overloaded with patients requiring emergent treatment, and there are higher rates of infectious wound, gastrointestinal, and respiratory diseases in the immediate aftermath [66]. Similarly, the rates of psychiatric conditions like major depressive disorder and post-traumatic stress disorder also increases in natural disaster survivors [67–69]. Current literature on the impact of natural disasters on cancer care is limited, especially on its impact on childhood cancer care. However, available studies have shown that interruptions and delays in cancer treatment led to worsened disease control, less disease-free survival years, and early death [70, 71]. Furthermore, natural disasters impose great difficulties as healthcare infrastructures may become compromised, with the destruction of medical records and biopsy specimens, and delayed elective imaging or diagnostic procedures [72].

### National cancer control programs (NCCPs)

A NCCP is a wide-ranging set of cancer control activities undertaken in a country to address their nation’s cancer burden. Developing and implementing an evidence and resource-based NCCP is a crucial step for countries, and, when linked to strong governing mechanisms, has been shown to lead to better cancer outcomes [73]. Unfortunately, many NCCPs worldwide lack implementation due to underfunding, lack of political will, or lack of expertise [74]. In the EMR, many inequalities exist in the presence and implementation of NCCPs [75]. 55% have standalone NCCPs, while 27% have non-communicable disease plans that include cancer, some of which are outdated. While 91% of these plans are fully or partially funded, the plans are not costed [75]. Similarly, among countries with established plans, only ten are operational, particularly those from LICs [75]. In a survey conducted by WHO EMR office in 2019, only six countries included childhood cancer in their priority benefits package [8].

### Moving forward

Countries in the EMR continue to face serious challenges in maintaining efficient and well-functioning health systems and providing quality services to paediatric cancer patients. Due to past and ongoing geo-political, economic, and natural events growth in capacity for caring for children with cancer has been hampered in many of the regional countries; in fact, in some countries this has resulted in a decline in the ability to provide care. On the other hand, significant enhancement has occurred in the region, not only in HICs but also in many of the L/LMICs. Overall, we believe that the capacity to diagnose and treat paediatric cancer has improved considerably over the past decade. One recent impetus for this has been the WHO’s launch of the Global Initiative for Childhood Cancer (GICC) in 2018, which aims to improve overall childhood cancer survival to 60% by 2030 [76]. In 2020, WHO-EMR awarded seed grants for projects in six countries to spearhead the process of improvement. Two EMR countries, Morocco as a pilot and recently Pakistan, have been identified as focus countries where WHO will help assess, plan, implement, and monitor the state of paediatric oncology holistically. It is expected that the participation of the governments in these and other countries as key stakeholders will enhance the development and implementation of national cancer control plans, develop comprehensive care strategies, and improve access to paediatric oncology care.

Enough diversity exists within the region to support locoregional collaboration and twinning, especially as language and cultural background represent commonalities that make it more feasible. The POEM group was established in 2013 with this goal in mind [77]. Consisting of healthcare professionals from the region, over the past few years the POEM group has established a regional forum for tumour boards for patient care discussions, has conducted nursing workshops, and scientific symposia for education in paediatric oncology. It has also sponsored the training of seven paediatric oncologists at well-established regional programs and is now embarking on regional, multi-national, clinical trials.

**Figure 2.**
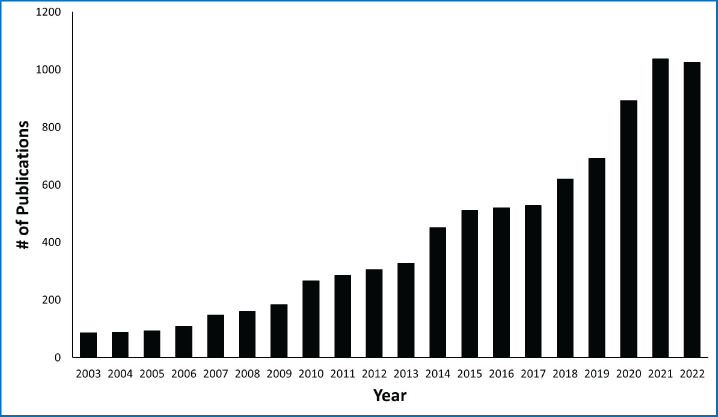
Number of scientific papers related to childhood cancer published annually from the EMR countries (Data from PubMed).

Activity towards improvements in childhood cancer care is also reflected in the regional scientific output. Since 2003 the annual number of publications on PubMed^R^ relating to childhood cancer from the regional countries has increased more than ten-fold (Figure 2). This not only indicates a greater number of people contributing to this but also more investment of time and money in scientifically documenting care quality.

Regional progress towards improvement in the care of children with cancer has been sound, however much work needs to be done. Documentation of the magnitude of the problem requires establishment of population-based registries. Training of personnel with the provision of centres for them to work in is needed in many countries to serve a large numbers of patients. While referral systems are beginning to be established in some HICs, more such mechanisms are required to ensure timely access to diagnosis and care. Programs need to be developed to curtail patient-incurred costs. Work on all of this has begun in the EMR and needs to be supported for sustained improvements in the care of children with cancer.

## Conclusion

The EMR continues to be one of high variability, with countries and childhood cancer care facilities that have moved far in their care quality continuum adjacent to others that are seriously deficient. Natural and human disasters have afflicted many of the regional countries and have hampered or disrupted their healthcare progress. Much work needs to be done at national levels, with ownership and responsibility taken by relevant health ministries, to enhance awareness, early recognition and referral, and access to affordable care. Progress on these indicators must be documented through the collection and analysis of population level data from national childhood cancer registries, that are insufficiently implemented in the region. However, considerable focus is now being placed on childhood cancer care in the region, particularly following the implementation of the GICC. Intra-regional, international collaborative efforts are underway that are utilising the existing resources within the EMR to support capacity enhancement and care quality improvements. Legislated governmental action is being seen. Continuous effort and persistence have started to bear fruit, and this region has the potential to demonstrate significant improvements in the care of children with cancer in the coming years.

## Conflicts of interest

The authors have no financial or non-financial conflicts of interest to declare.

## Funding declaration

No external funding was utilised for this project.

## Figures and Tables

**Table 1.** Country information regarding demographics and paediatric cancer.

Country	World Bank income status [3]	Population (millions) [78]	Proportion 0–14 years old (%) [78]	>25% pop. below the international poverty line [5]	CIR^b^ for childhood cancer per 100,000 population^a^ [78]	Annual number of paediatric cancer cases^a^ [78]	Childhood Cancer mortality^a^ [78]	Cancer registration [18]
Afghanistan	LIC	42.2	43	Yes	8.3	1,349	752	None
Bahrain	HIC	1.5	20	No	8.4	26	3	National
Djibouti	LMIC	1.1	30	No	7.4	21	14	None
Egypt	LMIC	112.7	33	Yes	12.0	4,181	1,581	Subnational
Iran	LMIC	89.2	23	No	13.7	2,841	1,290	National
Iraq	UMIC	45.5	37	No	10.5	1,589	602	Subnational
Jordan	UMIC	11.3	32	No	9.9	333	110	National
Kuwait	HIC	4.3	20	No	11.2	103	25	National
Lebanon	LMIC	5.4	27	No	12.5	214	65	National
Libya	UMIC	6.9	28	No	11.1	212	67	Subnational
Morocco	LMIC	37.8	26	No	12.7	1,252	487	Subnational
OPT	LIC	5.4	38	No	10.2	200	76	None
Oman	HIC	4.6	27	No	11.8	135	27	National
Pakistan	LMIC	240.5	36	Yes	7.2	5,574	2,769	Subnational
Qatar	HIC	2.7	16	No	8.1	32	3	National
Saudi Arabia	HIC	36.9	26	No	12.5	1,076	246	National
Somalia	LIC	18.1	47	No	10.8	789	527	None
Sudan	LIC	48.1	41	Yes	9.2	1,604	846	None
Syrian Arab Republic	LIC	23.2	30	Yes	10.6	572	251	National
Tunisia	LMIC	12.5	25	No	11.1	320	96	Subnational
UAE	HIC	9.5	15	No	11.0	161	28	Subnational
Yemen	LIC	34.4	39	No	10.9	1,263	670	Subnational

a All data are for children aged 0–14 years and for 2020;

b CIR = Crude Incidence Rate

LIC = Low Income Countries; LMIC = Lower-Middle Income Countries; UMIC = Upper-Middle Income Countries; HIC = High Income Countries
